# Chemoenzymatic synthesis of unusual antibody glycoforms using *E. coli* expressed human and bovine β-1,4-galactosyltransferase and their mutants

**DOI:** 10.1016/j.carres.2026.110019

**Published:** 2026-06-26

**Authors:** D. Natasha Owitipana, Darnell Harris, Helena Yun, Guanghui Zong, Lai-Xi Wang

**Affiliations:** Department of Chemistry and Biochemistry, University of Maryland, 8051 Regents Drive, College Park, MD, 20742, United States

**Keywords:** Antibody glycoforms, β-1,4-galactosyltransferase, Chemoenzymatic synthesis, *E. coli* expression, Sialyltransferase, Siglec-2, LacdiNAc

## Abstract

Antibody Fc glycosylation critically influences effector functions, motivating the development of chemoenzymatic strategies to generate homogeneous glycoforms. However, limited availability and high cost of mammalian glycosyltransferases remain to be significant constraints. Here, we report the high-yield expression of human and bovine β-1,4-galactosyltransferases (B4GalT1) and their single-point mutants (Y285L and Y289L, respectively) as maltose-binding protein fusions in *Escherichia coli*. A comparative analysis revealed that human B4GalT1 and its Y285L mutant exhibited substantially higher activity than bovine enzymes in transferring galactose (Gal) and N-acetylgalactosamine (GalNAc) from the corresponding UDP-sugar to terminal N-acetylglucosamine residues on Fc N-glycans of intact antibodies. These enzymes enabled the synthesis of antibody glycoforms bearing GalNAcβ1-4GlcNAc (LacdiNAc) in place of LacNAc. An unexpected finding was that the α-2,6-sialyltransferase from *Photobacterium damselae* (Pd2,6ST) and the α-2,3-sialyltransferase mutant PmST1 M144D from *Pasteurella multocida* recognized terminal GalNAc, but not Gal, in the intact antibody for sialylation. Interestingly, sialylation of terminal Gal was restored Pd2,6ST for the trastuzumab variant carrying a point F241A mutation. These results suggest that the intramolecular interaction between the terminal galactose and aromatic residues such as F241 in the Fc domain may hinder the enzymatic sialylation, while the F241A mutation partially releases such an interaction thus enhancing the sialylation efficiency. Receptor binding analysis showed that the antibodies carrying a LacdiNAc moiety exhibited comparable affinity to the FcγIIIA receptor and C1q protein as the LacNAc terminated glycoform, while the sialylated LacdiNAc glycoform could still bind to Siglec-2, albeit at lower affinity than the common sialylated LacNAc glycoform. These results expand the enzymatic toolkit for antibody glycoengineering and highlight the functional impact of LacdiNAc incorporation.

## 1. Introduction

Glycosylation is a ubiquitous posttranslational modification of proteins that plays an important role in modulating protein’s structure and functions [1,2]. Monoclonal antibodies (mAbs) are a class of therapeutic glycoproteins that are widely used for the treatment of human diseases including cancer, infectious diseases, and autoimmune disorders [3–5]. Most therapeutic antibodies belong to the immunoglobulin G (IgG) subclass, which carry a conserved biantennary complex type N-glycan at the Asn-297 site in its Fragment crystallizable (Fc) domain. Compelling data have shown that the structures of the conserved Fc glycans have profound effects on antibody’s effector functions, such as antibody-dependent cellular cytotoxicity (ADCC), antibody-dependent cell-mediated phagocytosis (ADCP) and complement dependent cytotoxicity (CDC) [6,7]. For example, the presence of core fucose, which is α1,6-linked to the innermost N-acetylglucosamine moiety of the Fc glycans, significantly decreases ADCC activity by reducing antibody’s affinity for FcγRIIIA, whereas terminal sialylation has been implicated in modulating ADCC and anti-inflammatory responses [8–12]. On the other hand, terminal galactosylation of the Fc glycans also plays an important role in modulating antibody activities, leading to enhanced ADCC and CDC activities [13,14]. Structural studies have shown that terminal galactose residues can interact intramolecularly with residues such as F241 and/or F243 on the Fc CH2 domain [15], which restricts glycan flexibility but also constrains CH2 domain mobility, thereby reducing the entropic penalty associated with FcγIIIA receptor (FcγRIIIA) binding. This pre-organization of Fc conformation results in enhanced affinity of antibodies for Fcγ receptors and C1q complement protein [13,16–18]. However, a major challenge in studying antibody functions comes from the structural microheterogeneity of Fc glycosylation. Natural and recombinant antibodies are usually produced as mixtures of heterogeneous glycoforms, from which structurally defined and homogeneous glycoforms are difficult to isolate by common chromatographic techniques [19]. To overcome this challenge, biosynthetic pathway engineering and *in vitro* chemoenzymatic glycoengineering have been developed to generate structurally well-defined antibody glycoforms [20–22]. Chemoenzymatic glycoengineering employing glycosyltransferases and glycosidases for *in vitro* glycan editing has emerged as a powerful and versatile strategy for remodeling antibody glycans. This approach can provide not only homogeneous natural glycoforms, but also site-selectively modified glycoforms for structural and functional studies [10,21–27].

Efficient *in vitro* Fc glycan remodeling relies on the availability of active glycosyltransferases (GTs) and/or endoglycosidases. β-1,4-Galactosyltransferases are a family of enzymes in vertebrates (B4GalT1–7), which catalyzes the transfer of galactose (Gal) from UDP-Gal to *N*-acetylglucosamine (GlcNAc) acceptor in a β-1,4-glycosidic linkage [28]. In 2002, Qasba and co-workers performed structure-based design and reported that a novel single mutant of bovine B4GalT1, Y289L, showed a broadened donor substrate specificity, enabling the transfer of N-acetylgalactosamine (GalNAc) from UDP-GalNAc to a GlcNAc acceptor with high efficiency while retaining the galactosyltransferase activity [29]. Moreover, it has been demonstrated that the mutation of Tyr to Leu expands the binding site of the donor sugar thus allowing the recognition of the C2-modified galactose such as N-azidoacetylgalactosamine (GalNAz) for further conjugation, enabling the synthesis of site-specific antibody-drug conjugates [30–34]. While *E. coli* is biotechnology’s work horse for protein expression because of its low cost, ease of handling and relatively high yield, *E. coli* expression of mammalian enzymes, particularly glycosyltransferases, remains a challenging task [35]. Bovine B4GalT1-Y289L mutant (bB4GalT1-Y289L) has been expressed in *E. coli* as inclusion bodies, which require refolding to obtain the soluble active enzyme [36,37]. Recently, Chen and co-workers have expressed several mammalian glycosyltransferases, including bB4GalT1, as maltose binding protein (MBP)-fusion enzymes in *E. coli* Origami B strain as soluble proteins in high yield [38]. Inspired by this promising result, we report in this paper the expression of both bovine and human B4GalT1 and their mutants in engineered *E. coli*, their comparative activity, and their application for making unusual antibody glycoforms.

We found that both human and bovine B4GalT1 and their point mutants (Y285L/Y289L) could be successfully expressed in an engineered *E. coli* system in excellent yield as N-terminal maltose binding protein (MBP) fusion enzymes. Comparative analysis showed that the human enzymes are significantly better than the bovine counterparts at transferring galactose or its derivative (GalNAc) directly to antibody Fc N-glycans. Utilizing the mutant enzymes, we were able to make antibody Fc glycoforms carrying a GalNAcβ1,4GlcNAc (LacdiNAc) motif. It has been previously reported that certain mammalian glycoproteins carry LacdiNAc structural moieties in their N-glycans [39,40], and the LacdiNAc epitope can be sulfated [41–45] or sialylated [46–49]. However, it has not been reported if natural antibodies carry such a structural motif. Thus, we investigated the substrate specificity of two bacterial sialyltransferases on the unique epitope, LacdiNAc, in the context of an antibody. We found that while the α-2,6 sialyltransferase from *Photobacterium damselae* (Pd2,6ST) and the α-2,3 sialyltransferase from *Pasteurella multocida* (PmST1 M144D) were unable to transfer a sialic acid moiety to the common galactose-terminated Fc glycan linked to trastuzumab (a therapeutic monoclonal antibody), surprisingly, the two enzymes could sialylate the unusual GalNAc-terminated Fc glycan in intact trastuzumab, enabling the synthesis of the novel sialylated antibody glycoforms. Moreover, we found that a single F241A mutation at the Fc domain of trastuzumab could partially rescue the sialylation of galactose-terminal antibody glycoform by the bacterial enzymes. Finally, binding studies indicated that the antibodies carrying LacdiNAc- and LacNAc glycans showed comparable affinity to the FcγRIIIA receptor, while the novel sialylated LacdiNAc glycoform could bind to Siglec-2, albeit at lower affinity than the common sialylated LacNAc antibody glycoform.

## 2. Results and discussion

### 2.1. Expression of human and bovine β1,4-galactosyltransferase 1 (B4GalT1) and their mutants

**Fig. 1.**
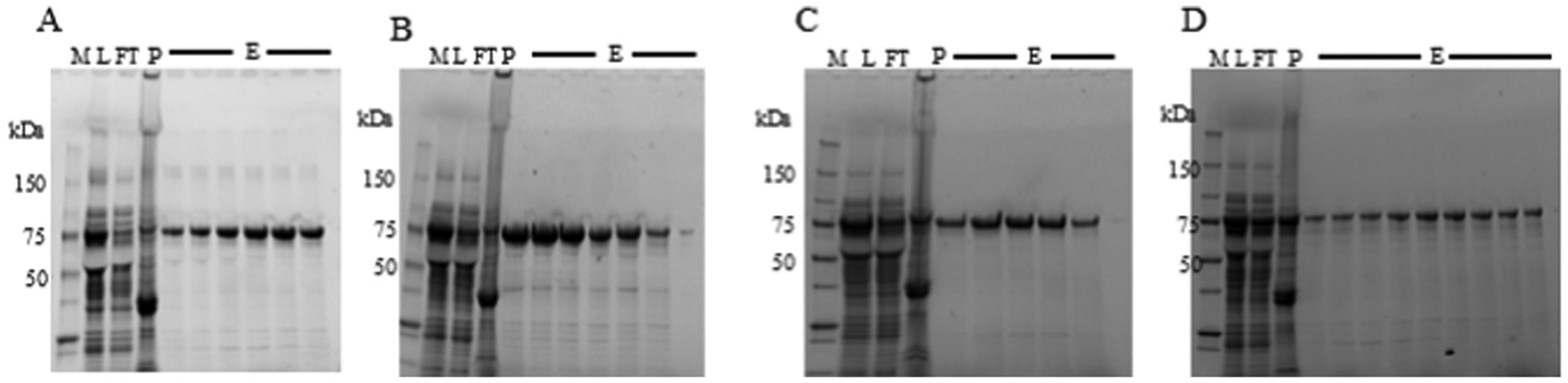
SDS-PAGE analysis of the expression and purification of hB4GalT1 and bB4GalT1 WT and their mutants in *E. coli* Origami B (DE3) as N-terminal MBP fusions. A) MBP_Del1_43 hB4GalT1_His6. B) MBP_Del1_43 bB4GalT1. C) MBP_Del1_43 hB4GalT1-Y285L_His6. D) MBP_Del1_43 bB4GalT1-Y289L_His6. Lanes: M, Precision Plus Protein^™^ Standard (250 – 10 kD); L, supernatant after lysis; FT, flowthrough from HisTrap^™^ HP 5 ml column; P, pellet after lysis; E, elution fractions containing the enzyme.

B4GalT1 is a principal enzyme used in chemoenzymatic methods as it catalyzes the formation of Galβ1,4GlcNAc by transferring Gal from UDP-Gal to an accepting GlcNAc, forming galactosylated complex type N-glycans. Bovine B4GalT1has been expressed in *E. coli* as inclusion bodies, which must be refolded to provide a soluble active enzyme [36]. The catalytic domain of human B4GalT1 has also been expressed in *E. coli*, but the yield was not reported [50]. More recently, Chen and co-workers have successfully expressed bovine B4GalT1 in *E. coli* as an MBP-fusion enzyme [38]. By following Chen’s approach, we sought to explore the expression of both human and bovine B4GalT1 and their mutants in an engineered *E. coli* as an MBP fusion and compare their enzymatic activities for synthesizing different antibody glycoforms. Thus, we generated plasmids for both human and bovine B4GalT1 by fusing the MBP to the N-terminus, with the transmembrane components being removed, and having a six His-tag at the C-terminal for Ni^2+^ affinity-based purification. In contrast to the previous design, we chose to keep the stem region with the catalytic domain. Retaining the stem region was shown to increase folding efficiency of human and bovine B4GaT1 when the enzymes were refolded from inclusion bodies [37]. We found that the two constructs, human MBP_Del1_43 B4GalT1_His6 (referred hereafter as hB4GalT1) and bovine MBP_Del1_43 B4GalT1_His6 (referred hereafter as bB4GalT1), were expressed successfully in *E. coli* Origami B (DE3) cells harboring pGro7 chaperone, producing the corresponding soluble enzymes in high yield (Fig. 1A and B, Table 1). We also generated single point mutants for both bB4GalT1-Y289L and hB4GalT1-Y285L via site-directed mutagenesis. We found that the mutant enzymes could be expressed in comparable yields as the wild type enzymes in *E. coli*. (Fig. 1C and D–Table 1). It should be mentioned that this is the first report of the production of hB4GalT1-Y285L, bB4GalT-Y289L in *E. coli*.

### 2.2. Evaluation of the activity of human and bovine B4GalT1 enzymes for transferring galactose (Gal) moieties to antibodies

**Fig. 2.**
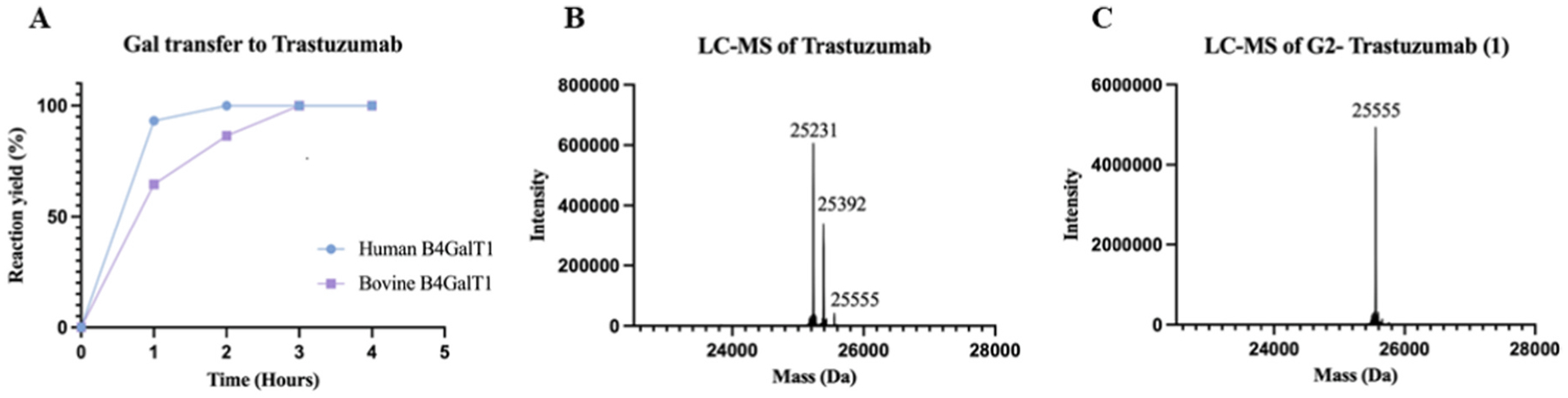
Comparison of *E. coli* expressed human and bovine B4GalT1 in producing G2-trastuzumab glycoform. A) Time course profile of the hB4GalT1-and bB4GalT1-catalyzed Gal transfer from UDP-Gal to trastuzumab (n = 1). B) Charge deconvoluted LC-MS of the Fc domain of native trastuzumab after IdeS treatment (G0: expected 25,229.7 Da, found 25,231.0 Da; G1, expected 25,391.7 Da, found 25,392.0 Da; G2, expected 25,553.8 Da, found 25,555.0 Da). C) Charge deconvoluted LC-MS of the Fc domain of G2-trastuzumab (**1**) after IdeS treatment (expected, 25,553.8 Da; found, 25,555.0 Da) Enzymatic reaction conditions: A mixture of trastuzumab (25 mg/ml), UDP-Gal (15 eq), and hB4GalT1 or bB4GalT1 (5%, w/w, enzyme: antibody) was incubated in a buffer containing 10 mM MnCl 2 (pH 7.4) at 37 °C. The reactions were monitored by LC-MS analysis of the Fc fragment released by IdeS treatment of the antibodies.

**Scheme 1.**
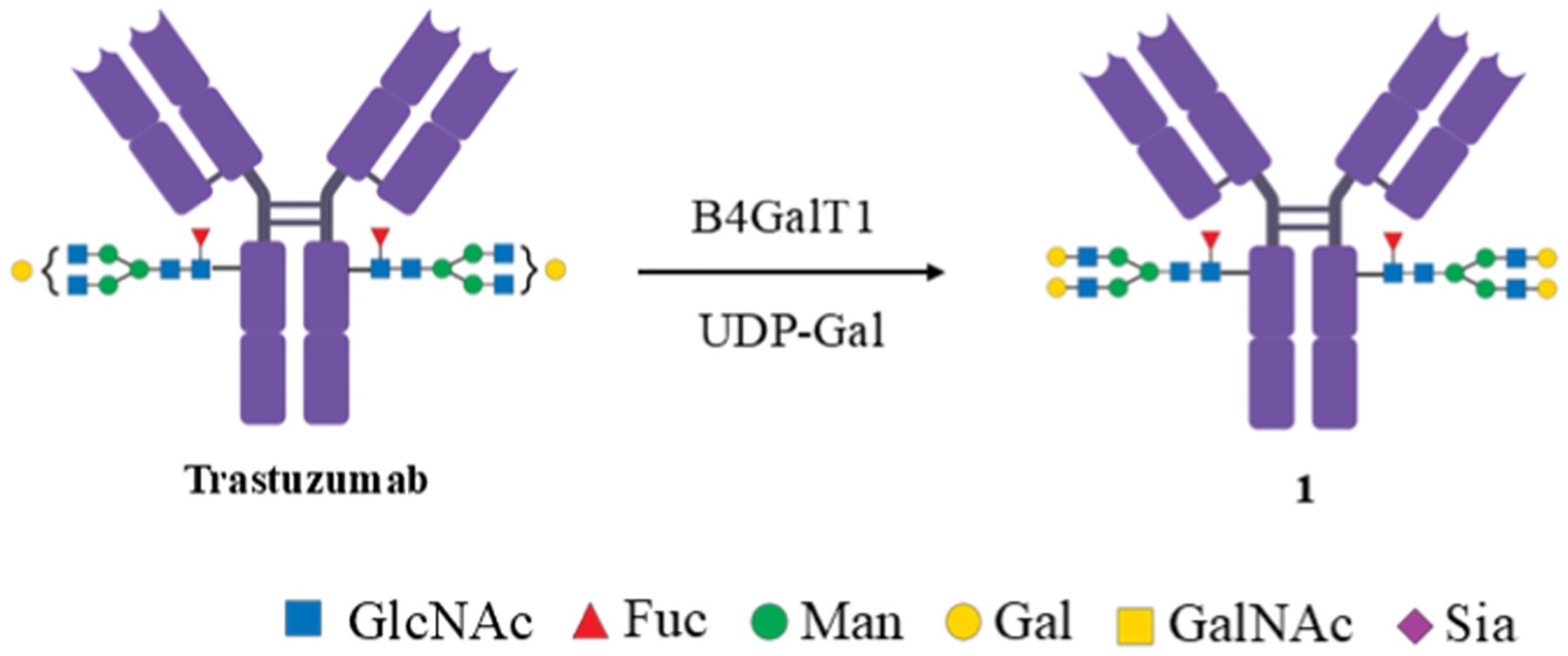
Enzymatic Fc glycan remodeling of trastuzumab to make the homogenous G2-glycoform using *E. coli* expressed B4GalT1.

To test and compare the activities of the *E. coli*-expressed recombinant B4GalT1, we chose trastuzumab, a therapeutic monoclonal IgG1 antibody as the substrate. The trastuzumab expressed in-house using HEK293T cells was a mixture of G0F, G1F, and G2F glycoforms, where 0, 1, and 2 indicate the number of terminal galactoses on the Fc N-glycans and F indicates core fucosylation, as revealed by LC-MS analysis (Fig. 2B). We found that both human and bovine B4GalT1 were active and could transfer Gal to trastuzumab from UDP-Gal (Scheme 1) (Fig. 2A). Nevertheless, it was observed that the recombinant human enzyme, hB4GalT1, was much more efficient than the bovine enzyme (bB4GalT1) at converting the heterogeneous population to a homogeneous G2 glycoform (**1**) of trastuzumab (Fig. 2C). Under the same conditions indicated, the hB4GalT1 produced the fully galactosylated homogeneous G2 glycoform within 1 h, while the bB4GalT1 catalyzed reaction took 3 h to complete the transformation. All reactions were done using 5% (w/w) of the enzyme, with 15 eq of UDP-Gal donor and 10 mM of MnCl_2_. Excess UDP-Gal was used to accelerate the galactosylation reaction. This result signifies the efficient production of the therapeutically relevant G2-glycoform *in vitro* by the *E. coli* expressed mammalian enzymes as a maltose binding protein (MBP)-fusion protein. It should be mentioned that MBP fusion has been used as a solubility enhancing tag for the expression of otherwise insoluble proteins in *E. coli* [51]. Depending on the nature of the fused protein some proteins are shown to fold spontaneously, while other proteins require the assistance of a chaperone [52]. In our efforts, we expressed both human and bovine B4GalT1 enzymes in *E. coli* with and without the pGro7 chaperone. We observed that in the absence of the chaperone, the yield and activity of the enzymes were lower than those with chaperone co-expression, suggesting that the B4GalT1 expression requires the chaperone for folding.

### 2.3. Evaluation of the activity of human and bovine B4GalT1 mutants for transferring GalNAc moieties to antibodies

**Fig. 3.**
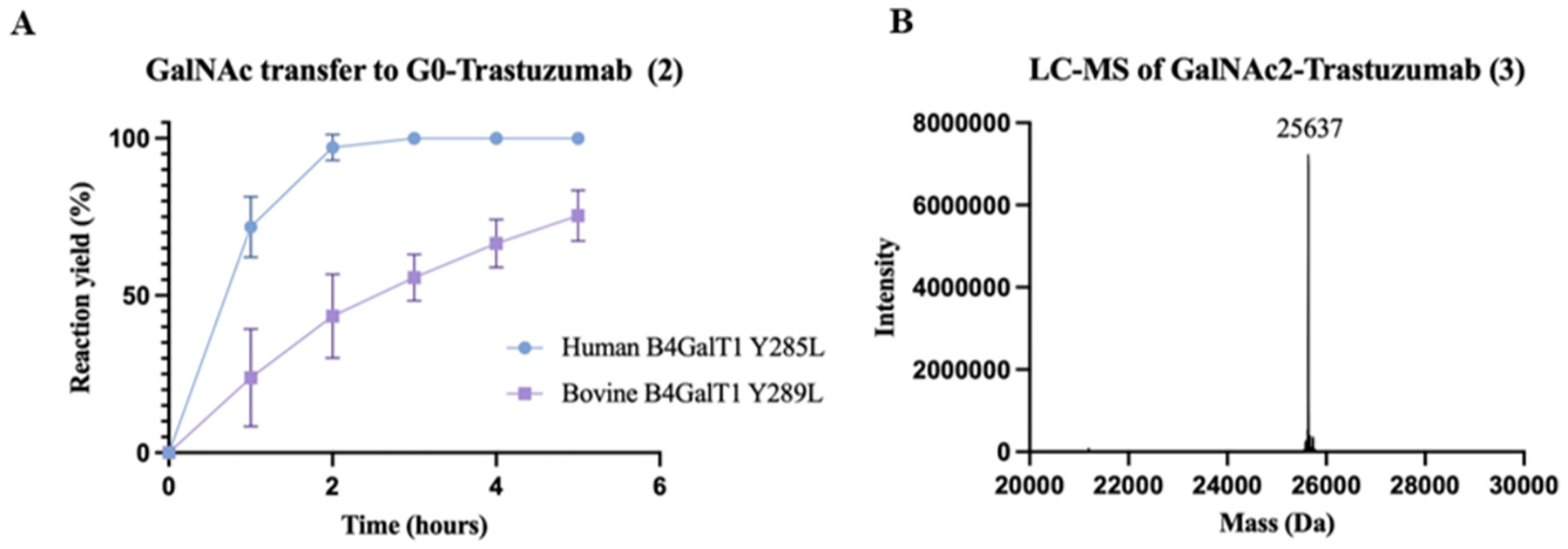
Comparative analysis of the *E. coli* expressed human and bovine B4GalT1 mutants for transferring GalNAc to G0 antibody glycoform. A) time course profile of the hB4GalT1-Y285L and bB4GalT1-Y289L catalyzed transfer of GalNAc to G0 antibody glycoform (**2**) to yield the LacdiNAc trastuzumab (**3**) (n = 2). B) Charge deconvoluted LC-MS of the Fc domain of the GalNAc2-trastuzumab (**3**) after IdeS treatment (expected, 25,635.7 Da, found 25637.0 Da) Enzymatic reaction conditions: A mixture of the G0-trastuzumab (**2**) (15 mg/ml), UDP-GalNAc (10 eq), and hB4GalT1-Y285L mutant or bB4GalT1-Y289L mutant (%, w/w, enzyme: antibody) was incubated in a buffer containing 10 mM MnCl_2_ (pH 7.4) at 37 °C. The reactions were monitored by LC-MS analysis of the Fc fragment released by IdeS treatment of the antibodies. Data are presented as mean ± s.d., *n*=*2* biological replicates.

**Scheme 2.**
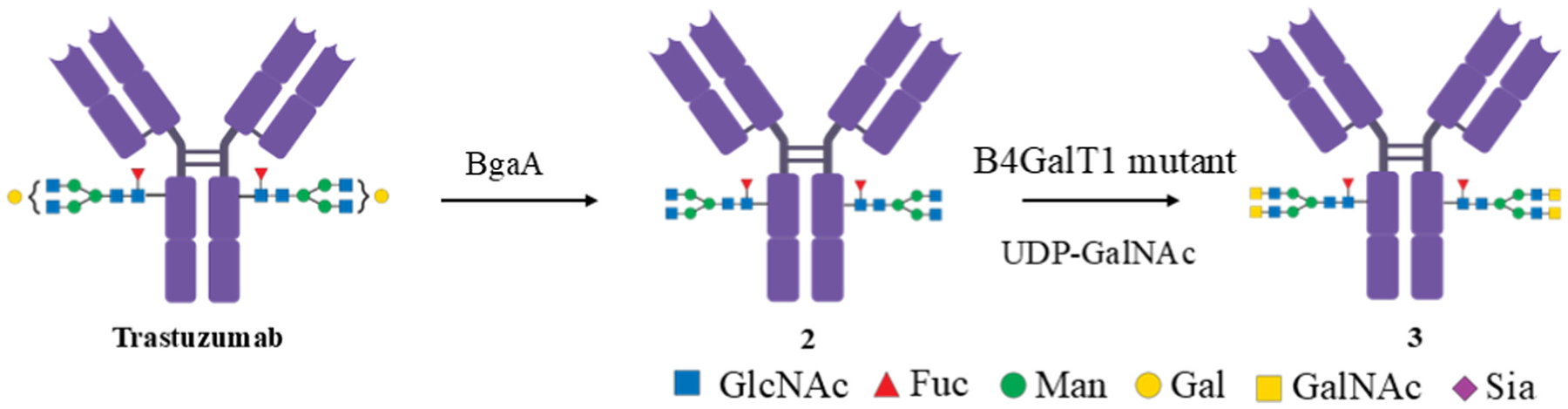
Enzymatic Fc glycan remodeling of trastuzumab to synthesize LacdiNAc terminated antibody glycoform using *E. coli* expressed B4GalT1 mutants.

Activity of the mutant enzymes, hB4GalT1-Y285L and bB4GalT1-Y289L were evaluated by the transfer of GalNAc from UDP-GalNAc to G0-Trastuzumab (Scheme 2). The agalactosylated glycoform (G0-Trastuzumab, **2**) was produced by treating trastuzumab with BgaA, a β-1,4-galactosidase from *Streptococcus pneumoniae*. **2** contains four GlcNAcs per antibody molecule as acceptor substrates for B4GalT1. The enzymatic reactions were performed at 37 °C, using 5% (w/w) of the enzyme, with 10 eq of UDP-GalNAc donor and 10 mM of MnCl_2_, following the previously published procedures [53]. We observed that the GalNAc transfer to **2,** catalyzed by hB4GalT1-Y285L, was completed in 2 h under the condition, whereas the reaction catalyzed by bB4GalT1-Y289L gave less than 50% product at 2 h (Fig. 3A and B). This result indicates again that the human B4GalT1 mutant is superior to the bovine mutant enzyme for transferring GalNAc to intact antibody. High efficiency of the enzyme combined with the ease of its production in the *E. coli* platform makes the hB4GalT1 WT and Y285L mutant an efficient tool for chemoenzymatic antibody glycoengineering. While some mammalian glycoproteins have been previously reported to carry LacdiNAc structural moieties in their N-glycans [39–49,54–56], their presence in natural or recombinant antibodies has not been described. Thus, an efficient synthesis of LacdiNAc-containing antibody glycoforms allows an opportunity to study their potential functions.

### 2.4. Synthesis of sialylated LacdiNAc antibody glycoforms

**Fig. 4.**
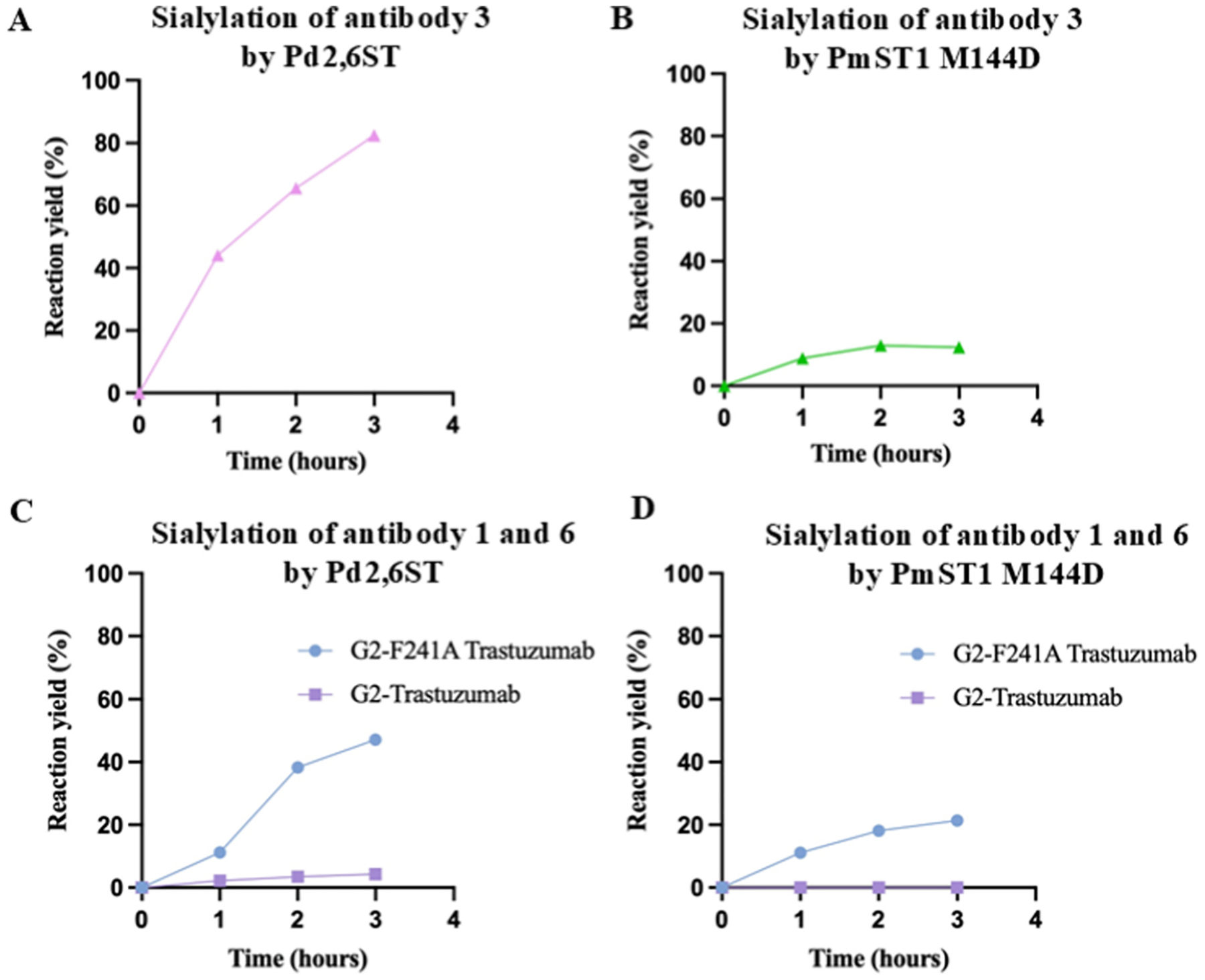
Comparative analysis of enzymatic sialylation of antibodies by the bacterial sialyltransferases. A) Time course profile of sialylation of LacdiNAc-trastuzumab (**3**) catalyzed by Pd2,6ST (n = 1). B) Time course profile of sialylation of **3** catalyzed by PmST1 M144D (n = 1). C) Comparison of Pd2,6ST catalyzed sialylation of G2-trastuzumab glycoform (**1**) and G2-F241A trastuzumab (**6**) (n = 1). D) Comparison of PmST1 M144D catalyzed sialylation of **1** and **6** (n = 1). Enzymatic reaction conditions: A mixture of the LacdiNAc-trastuzumab (**3**), the G2-trastuzumab (**1**), or the G2-F241A trastuzumab **(6)** (30 mg/ml), and CMP-Neu5Ac (150 eq) in a Tris buffer (100 mM, pH 7.5) was incubated with Pd2,6ST or PmST1 M144D (0.2 mg/ml) at 37 °C. The reactions were monitored by LC-MS analysis of the Fc fragment released by IdeS treatment of the antibodies. The reaction yield was calculated as the sum of the disialylated product yield and one-half of monosialylated product yield.

**Scheme 3.**
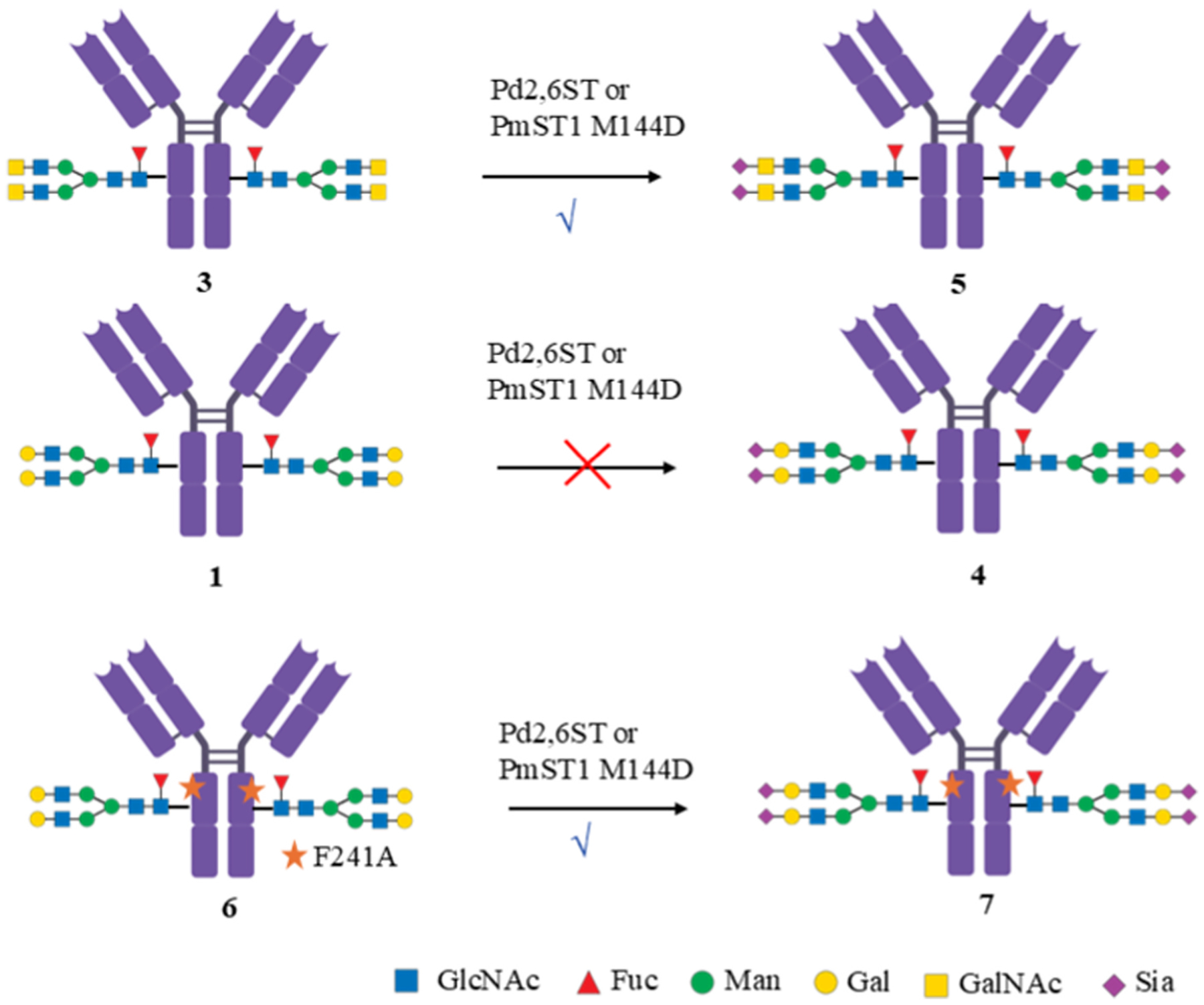
Enzymatic Fc glycan remodeling to produce sialylated antibody glycoforms using bacterial sialyltransferases (Pd2,6ST and PmST1 M144D).

The presence of Neu5Acα2,6GalNAcβ1,4GlcNAc has been reported for certain glycoproteins such as members of the prolactin/growth hormone family [47], human protein C (rHPC) [46], and bovine lactotransferrin [57]. However, it is not clear how different sialyltransferases act on the terminal LacdiNAc moiety in N-glycans. Thus, we first evaluated the ability of two bacterial sialyltransferases to sialylate the free N-glycan carrying terminal GalNAc moiety, including the α-2,6-sialyltransferase from *Photobacterium damselae* (Pd2,6ST) [58,59], and the α-2,3-sialyltransferase mutant derived from *Pasteurella multocida* (PmST1 M144D) [60]. A GalNAc-terminated biantennary N-glycan (GalNAc2-GN2) was synthesized by transferring GalNAc moieties to the G0-GN2 glycan with hB4GalT1-Y285L mutant, which was used as the acceptor substrate to test the two bacterial sialyltransferases (Scheme S1). The enzymatic reactions were monitored by HPAEC-PAD analysis. It was found that Pd2,6ST could sialylate the GalNAc-terminated free N-glycan efficiently and completed the reaction within 45 min under the condition (Fig. S1), while the reaction catalyzed by the α-2,3 sialyltransferase (PmST1 M144D) was much slower, and only ca. 50% sialylation could be achieved after pushing with more enzyme and with a longer reaction time (48 h) (Fig. S2). Next, we tested the sialyltransferases on the Fc N-glycans in the context of antibody. We found that Pd2,6ST could perform efficient sialylation on the LacdiNAc Fc glycan of the intact antibody (**3**) to give the unusual sialylated LacdiNAc glycoform (**5**) (Fig. 4A). The LC-MS analysis was shown in Fig. S3A. Another bacterial sialyltransferase, PmST1 M144D could also sialylate the LacdiNAc Fc glycan of the intact antibody (**3**) to give an α-2,3-sialylated glycoform, but the reaction was much slower and gave less than 20% conversion after 3 h under the condition (Fig. 4B, LC-MS analysis was shown in Fig. S3B). To our surprise, both bacterial sialyltransferases (Pd2,6ST and PmST1 M144D) were unable to sialylate the common galactose-terminated Fc glycans of the intact antibody (Scheme 3, Fig. 4C and D). One possible explanation is that the terminal Gal and GalNAc in the intact antibody might be oriented differently so that the GalNAc could be more accessible to sialylation than the more common terminal galactose moiety. Indeed, previous structural studies have shown that the terminal Gal moieties of Fc glycans form extensive contacts with residues on the Fc CH2 domain including F241, F243, K246, E258 and T260. These glycan-protein interactions restrict the glycan’s flexibility and stabilize the Fc region which promotes enhanced effector molecule binding [15–17]. We hypothesized that these interactions may hinder access of sialyltransferases to the terminal Gal, whereas the uncommon terminal GalNAc may have less interactions with the Fc domain and thus is less restrained. To test this hypothesis, we generated the F241A mutant of trastuzumab, which has been shown to increase the accessibility of the antibody Fc N-glycan for sialylation by reducing Fc glycan and Fc domain interactions [61]. Consistent with our hypothesis, the bacterial sialyltransferase Pd2,6ST exhibited significantly improved sialylation of terminal Gal on trastuzumab-F241A (**6**) compared with G2-trastuzumab (**1**), and the PmST1 M144D sialyltransferase also showed some activity on the galactose-terminated G2-trastuzumab-F241A (**6**) (Fig. 4C and D). These findings confirm that the limited activity of the enzymes toward Gal in native trastuzumab is not due to an intrinsic inability of the enzyme to recognize Gal but rather results from restricted accessibility caused by intramolecular glycan–protein interactions within the Fc region. Notably, Pd2,6ST still displayed higher sialylation efficiency toward terminal GalNAc on native trastuzumab than toward Gal on trastuzumab-F241A, suggesting an inherent preference for GalNAc as an acceptor substrate.

### 2.5. Binding of the new synthetic antibody glycoforms with FcγRIIIA, C1q and Siglec-2

**Fig. 5.**
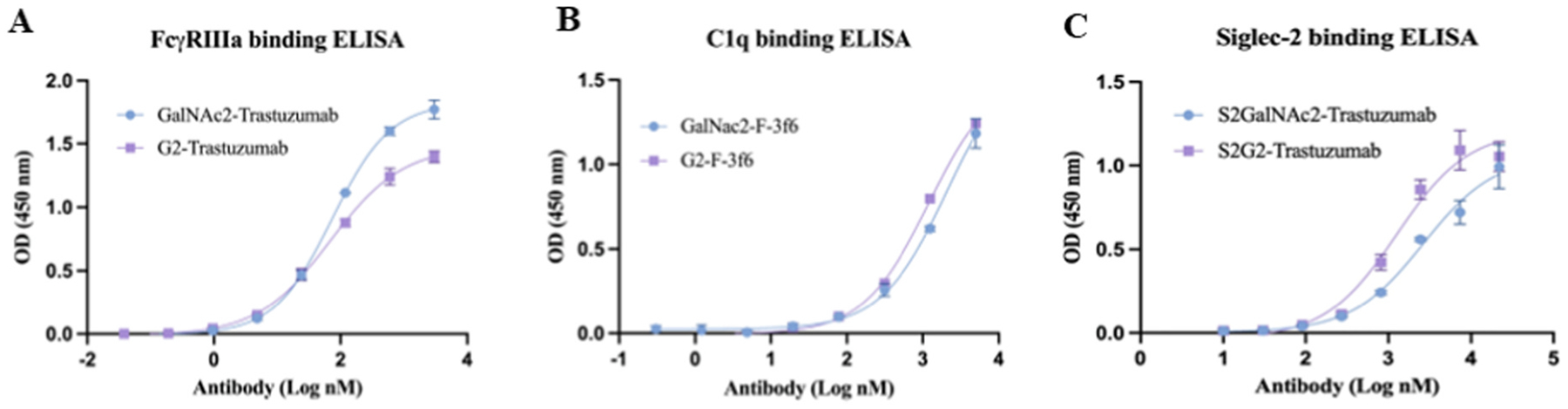
ELISA analysis of the binding of synthetic antibody glycoforms to their respective receptors. A) ELISA binding of trastuzumab glycoforms to FcγRIIIA. 0.5 μg/mL of FcγRIIIA V158 was coated in a 96 well plate overnight and allowed to bind with synthetic trastuzumab glycoforms in five-fold serial dilutions starting at a concentration of 3.0 μM. Observed EC_50_ values are 72 nM for GalNAc2-Trastuzumab (**3**) and 59 nM for G2-Trastuzumab (**1)**. B) ELISA based C1q binding to h3F6-IgG glycoforms. C1q (5 μg/mL) was coated in a 96 well plate overnight and allowed to bind with homogeneous GalNAc2-3F6 (**10**) and G2–3F6 (**8**) glycoforms in four-fold serial dilutions starting at a concentration of 5.0 μM. C) ELISA binding of trastuzumab glycoforms to Siglec-2. 2 μg/mL of Siglec-2 was coated in a 96 well plate overnight and allowed to bind with homogeneous sialylated trastuzumab glycoforms (**4** and **5**) in three-fold serial dilutions starting at a concentration of 22 μM). Data are presented as mean ± s.d., *n*=*3* biological replicates.

IgG induces antibody-dependent cellular cytotoxicity (ADCC) via its interaction with the Fcγ receptor IIIA (FcγRIIIA) on natural killer (NK) cells. Fine structures of the Fc N-glycan play a critical role in modulating the affinity of antibodies for FcγRIIIA. To assess the impact of the replacement of the common galactose moiety of the Fc glycoform (**1**) with the GalNAc moiety of the antibody glycoform (**3**), we performed ELISA analysis of the binding between FcγRIIIA-V158 and the antibody glycoforms (**1** and **3**). Our experimental data indicated that the two antibody glycoforms showed comparable affinity for the FcγIIIA receptor, with an EC_50_ being in the range of 60–70 nM (Fig. 5A). This result suggests that replacing the common galactose with an unusual GalNAc moiety in the Fc glycan does not significantly affect FcγRIIIA binding.

On the other hand, complement dependent cytotoxicity (CDC) is initiated by the binding of the antibody Fc to the C1q complement protein. This initial binding propagates the complement cascade leading to the formation of the membrane attack complex (MAC) and lysis of the target. Presence of galactose is shown to significantly increase the C1q binding and thereby CDC activity [13,14]. We have recently shown that selective 2- or 6-fluorination of the terminal galactose moiety in the Fc glycan of an antibacterial monoclonal antibody, 3F6-hIgG1, can enhance its complement protein C1q binding and bacterial killing activity [27]. The 3F6-hIgG1 antibody is a monoclonal antibody that binds and neutralizes Staphylococcal protein A (SpA) via activating the complement-dependent cytotoxicity (CDC) pathway [62]. The presence of galactose on the antibody 3F6-hIgG1 N-glycan was shown to significantly enhance its binding to C1q and thereby its CDC activity [14]. Thus, we ventured to assess if terminal GalNAc in the place of Gal might change the binding affinity of the 3F6-hIgG1 antibody to C1q. For this purpose, we synthesized the LacdiNAc glycoform of 3F6-hIgG1 using the hB4GalT1-Y285L enzyme for introducing the GalNAc moiety (Scheme S2). The identity and homogeneity of the product, the LacdiNAc glycoform of 3F6-hIgG1 (**10**) (Scheme S2) was confirmed by LC-MS analysis (Fig. S4). The G2 antibody glycoform of 3F6-hIgG1 (**8**) was synthesized by complete enzymatic galactosylation of the parent monoclonal antibody using hB4GalT1 and UDP-Gal (Scheme S2, Figure S5). Next, we performed ELISA analysis of the binding of complement protein C1q with the two antibody glycoforms. It was found that despite the modification of the terminal galactose with a GalNAc moiety, the two antibody glycoforms showed comparable affinity for C1q complement protein (Fig. 5B), indicating that the LacdiNAc 3F6 glycoform may have similar complement-dependent bacterial cell killing property.

Finally, we performed Siglec-2 binding of the novel sialylated LacdiNAc trastuzumab glycoform. Siglecs (sialic acid binding immunoglobulin-like lectins) are a class of glycan binding proteins and are cell surface receptors present mostly in the surface of immune cells that recognize self from non-self and initiate immune inhibitory response [63,64]. There are 14 members of the human Siglec receptor family and have distinct specificities in their recognition, such as the disaccharide sugar at the reducing end of the sialic acid, linkage, type of glycan and secondary modifications such as sulfation. Siglec-2 (CD22) is a lectin expressed on B-cells that regulates B-cell activation through inhibitory signals and plays a role in preventing autoimmunity. It has been reported that Siglec-2 recognizes and binds to Neu5Acα2,6GalNAc epitope [65]. We sought to characterize if Siglec-2 could recognize the sialylated LacdiNAc motif in the context of antibodies. We performed ELISA analysis of the two sialylated trastuzumab glycoforms (**4** and **5**) with Siglec-2. We found that the sialylated LacdiNAc glycoform (**5**) did bind to Siglec-2, although its affinity was a little reduced in comparison with the sialylated G2 glycoform (Fig. 5C). It will be interesting to evaluate if the usual sialylated LacdiNAc glycoforms are present in physiological or disease-associated antibodies.

## 3. Conclusion

This study establishes an efficient and scalable *E. coli* expression system for human and bovine β-4-galactosyltransferases (B4GalT1) and their corresponding mutants (Y285L and Y289L, respectively), overcoming a major bottleneck in enzyme availability for antibody glycoengineering. The superior catalytic activity of the human enzymes further highlights their value for the construction of well-defined antibody Fc glycoforms. Importantly, we demonstrate the feasibility of generating antibody glycoforms bearing LacdiNAc termini and uncover previously unrecognized substrate preferences of bacterial sialyltransferases, which preferentially sialylate GalNAc-terminated Fc glycans over their common Gal-terminated counterparts on intact antibodies. Using a single-point Fc mutant (F241A) that disrupts intramolecular glycan–protein interactions, we further show that the limited activity of bacterial sialyltransferases toward Gal-terminated Fc glycans in intact trastuzumab is primarily attributable to restricted glycan accessibility imposed by the Fc glycan–protein interactions. Functional studies reveal that LacdiNAc incorporation largely preserves FcγRIIIA and C1q binding, while subsequent sialylation modulates Siglec-2 recognition, underscoring the subtle yet significant biological consequences of Fc glycan remodeling. Collectively, these findings expand the enzymatic repertoire for precise Fc glycan modification and open new avenues for designing novel antibody glycoforms for tailored effector functions.

## 4. Experimental section

### 4.1. Materials

All chemicals and solvents were purchased from Sigma-Aldrich unless otherwise mentioned. Uridine 5′-diphospho-galactose (UDP-Gal), N-acetylneuraminic acid (Neu5Ac) and cytidine 5′-triphosphate disodium salt (CTP) were purchased from BIOSYNTH-Carbosynth. UDP-GalNAc was purchased from Midwest Bioprocessing center. His-Trap HP and HiTrap Protein A HP column from Cytiva. Origami^™^ B(DE3) Competent Cells – Novagen from Milipore Sigma. Chaperone set including pGro7 was purchased from Takara Bio USA. DH5α competent cells, BL21 (DE3) competent cells, Q5 site directed mutagenesis kit, restriction enzymes were purchased from New England Biolabs, Inc. QIAprep Spin Miniprep kit from QIAGEN. All sequencing was done by Psomagen. All primer and gene synthesis were done by GenScript. All primers for restriction site insertions were designed using NEBaseChanger offered by New England Biolabs Inc. All concentrations of DNA and proteins were determined by NanoDrop 2000c (Thermo Scientific). All plasmid extractions were done using QIAprep Spin Miniprep kit. C1q was purchased from Complement Technology, Inc. Siglec-2 (CD22) was purchased by Acro Biosystems. KPL TMB Microwell Peroxidase Substrate System was purchased from LGC sera care.

### 4.2. Methods

LC-MS analysis was performed on an Ultimate 3000 HPLC system coupled to an Exactive Plus Orbitrap mass spectrometer (Thermo Fischer Scientific) with C4 (whole antibody, gradient 5–95% aq MeCN containing 0.1% FA for 6 min, 0.4 mL/min) or C8 (IdeS digestion, gradient 25–35% aq MeCN containing 0.1% FA for 6 min, 0.4 mL/min) column. The mass spectra were deconvoluted using MagTran software.

HPAEC-PAD analysis was done on a Dionex^™^ ICS-6000 (Thermo Fisher Scientific) system with an electrochemical detector (ED50), and an anion exchange column (CarboPAC PA200 4 × 250 mm) for efficient separation of oligosaccharide isomers. Solvent lines consisted of line A (18.5 MilliQ water), line B (10 mM NaOH), line C (100 mM NaOH), and line D (1 M NaOAc in 100 mM NaOH). The method for free N-glycan analysis started with a 20-min isocratic run (0–20 min, 80% C, and 20% D), followed by linear gradient for 5 min (20–25 min, 50% C, and 50%D).

### 4.3. Gene constructs

The synthetic genes for Human B4GalT1 (GenBank: X14085.1), Bovine B4GalT1 (GenBank: X14558.1) were synthesized by GenScript Biotech. Briefly, gene sequences with transmembrane segments removed (1–43 AA in both human and bovine B4GalT1) were codon optimized for *E. coli* and encoded in the pMAL-c4X in frame with maltose binding protein (*Ava*I, BamH1) with an added 6xHis tag in the C-terminal.

Mutagenesis primers to change Y289L in Bovine B4GalT1 and Y285L in Human B4GalT1 were designed using NEBasechanger (v2.4.4) offered by NEB BioLabs Inc. The designed primers (F: 5′GTACGTGCAA ctgTTTGGTGGCGTTAGC3′, R: 5′GGCAGGCTAAAGCCG3′) were synthesized, and site directed mutagenesis was performed using Q5 site-directed mutagenesis kit following manufacture directions.

### 4.4. Enzyme expression and purification

Expression of the MBP-B4GalT1 in *E. coli* requires co-expression of pGro7 chaperone. Therefore *E. coli* Origami B (DE3) cells were double transformed with pGro7 and B4GalT1 plasmids. Origami B (DE3) cells were transformed with pGro7 plasmid and selected on Chloramphenicol resistance (0.017 mg/ml). Next, cells harboring the pGro7 cells were made competent using calcium chloride method. Briefly, 5 ml of LB (Chloramphenicol 0.017 mg/ml) was inoculated with *E. coli* Origami B (DE3) cells containing pGro7 and grown overnight. The overnight culture was added into a 100 ml LB broth (Chloramphenicol 0.017 mg/ml) and grown till OD_600_ nm reached ~ 0.35 – 4.0 and chilled on ice. The cells were harvested by centrifugation at 4300 rpm for 11 min at 4 °C. The harvested cell pellet was resuspended in 20 ml of ice-cold 50 mM CaCl_2_, incubated for 20 min on ice, and harvested at 4300 rpm for 11 min at 4 °C. The resulting cell pellet was resuspended in 2.5 ml of ice cold 50 mM CaCl_2_. A volume of 100 μL was used for transformation with 100 ng of B4GalT1 plasmid and the positive transformant were selected based on antibiotic resistance, Carbenicillin (0.1 mg/ml) for B4GalT1 and Chloramphenicol (0.017 mg/ml) for pGro7.

A 5 ml culture of *E. coli* Origami B (DE3) cells harboring pGro7 and B4GalT1 (Carbenicillin (0.1 mg/ml), Chloramphenicol (0.017 mg/ml)) was grown overnight at 37 °C by shaking. The 5 ml culture was inoculated into a 300 ml LB broth supplemented with Carbenicillin (0.1 mg/ml), Chloramphenicol (0.017 mg/ml) and l-arabinose (0.5 g/L) for chaperone expression. The culture was allowed to grow at 37 °C while shaking till OD_600 nm_ ~0.6. The culture was chilled at 16 °C for an hour and induced with IPTG (0.1 mM). After induction the culture was grown for 48 h at 16 °C shaking. The cells were harvested by centrifugation at 4500 rpm for 30 min at 4 °C.

The harvested cell pellet was resuspended in lysis buffer (50 mM Tris-HCl, pH 7.5300 mM NaCl, 20 mM imidazole). The resuspended cells were lysed by sonication (20% amplitude, 10s on/20s cooling, total on time 5 min). The lysate was centrifuged at 16,000 rpm for 30 min at 4 °C, the obtained supernatant was filtered before loading the column. The enzyme was purified using Ni^2+^ affinity chromatography. Briefly, the column was equilibrated with 5 column volumes (CVs) of binding buffer (50 mM Tris-HCl, pH 7.5, 300 mM NaCl, 20 mM imidazole). The filtered supernatant was loaded onto the column. For the elution process, washing was done for 10 CVs with binding buffer and gradient elution was done using elution butter (50 mM Tris-HCl, pH 7.5300 mM NaCl, 500 mM imidazole) starting from 4% to 100% over a span of 20 CV. Fractions containing the target enzyme was checked by SDS-PAGE and buffer exchanged to 50 mM-Tris-HCl, 250 mM NaCl, pH 7.5.

The sialyltransferases, an α-2,6 sialyltransferase from *Photobacterium damselae* (Pd2,6ST) and an α-2,3 sialyltransferase mutant derived from *Pasteurella multocida* (PmST1 M144D), were expressed as follows. Plasmids were transformed into *E. coli* BL21 (DE3) cells and grown in 300 ml of Luria-Bertani broth with Kanamycin (0.05 mg/ml) at 37 °C until an OD_600_ ~ 0.5. The culture was induced by isopropyl *β*-D-1-thio-galactosidase (0.1 mM) and allowed to grow at 16 °C for 24 h. The cells were harvested by centrifugation at 4500 rpm for 30 min. The enzymes were purified as described above using Ni^2+^ affinity chromatography.

### 4.5. Expression and purification of trastuzumab and trastuzumab-F241A mutant

The coding sequences of trastuzumab heavy and light chains were retrieved from the DrugBank online public database (DrugBank accession number - DB00072). Signal peptides for the two chains were used based on optimized sequences reported previously [66]. The heavy and light chains containing the N-terminal signal peptides MDWTWRF LFVVAAATGVQS and MDMRVPAQLLGLLLLWLSGARC, respectively, were inserted into separate pcDNA3.1 vectors using *Nhe*I/*Xho*I sites. The codon-optimized synthetic genes were purchased from GenScript.

Recombinant trastuzumab was produced by transient expression in HEK293T suspension cells. Cells were cultured in serum-free FreeStyle^™^ F17 Expression Medium at an initial density of 8 × 10^5^ cells/mL and maintained at 37 °C with 7% CO_2_ and shaking at 150 rpm. Transient transfection was carried out 20 to 24 h after seeding using plasmid DNA (heavy chain/light chain = 1:3, total 2 μg/mL) and polyethylenimine (PEI, 6 μg/mL). The cultures were continued under the same conditions for 6 d, with supplementation of l-glucose and GlutaMAX^™^ on day 4 to support cell viability. On day 6, the cultures were clarified by centrifugation (4500 rpm, 30 min, 4 °C), and the supernatant was purified by protein A affinity chromatography. Bound antibodies were eluted with 30 mM citrate buffer (pH 2.6), immediately neutralized with 1 M Tris-HCl (pH 8.5), and buffer-exchanged into PBS (pH 7.4). The trastuzumab-F241A mutant was constructed and expressed in the similar way, following the previously published procedures [61].

### 4.6. LC-MS analysis of antibody

For intact antibody analysis, the antibody sample was diluted in 0.1% formic acid in water at a final concentration of 0.05 mg/ml of antibody. An amount of 0.5 μg of antibody was injected to the system and the method used is described above.

For Fc domain analysis, the antibody was treated with IdeS to release the Fc. IdeS treatment was done by treating the antibody at 5% (w/w) enzyme: antibody and incubating at 37 °C for 10 min. The IdeS treated sample was quenched in 0.1% formic acid in water at a final concentration of 0.05 mg/ml of antibody. An amount of 0.5 μg of antibody was injected to the system and the method used is described above.

### 4.7. Activity assay for B4GalT1 enzymes

Transfer of Gal from UDP-Gal to trastuzumab catalyzed by Human and Bovine B4GalT1 were carried out simultaneously. Trastuzumab was buffer exchanged to 50 mM MOPS buffer (pH 7.4). The constituents were added at the final concentrations listed, trastuzumab (25 mg/ml), UDP-Gal (15 eq), Mn^2+^ (10 mM). The pH of the reactions was tested to be pH 7.4 The enzyme was added last at 5% (w/w) with enzyme:IgG. Time points were taken every hour up to 5 h and then overnight. All time points were treated with IdeS to release the Fc from the antibody and analyzed by LC-MS. Fc domain of G2-trastuzumab (**1**) after IdeS treatment, M = 25,553.8 Da, found: 25,555.0 Da.

GalNAc transfer was done on G0-trastuzumab (**2**). **2** was produced by treating trastuzumab with BgaA, a β1,4 galactosidase from *Streptococcus pneumoniae,* at a final enzyme concentration of 0.1 mg/ml ON at 37 °C. The production of **2** was confirmed by LC-MS analysis and purified using protein A chromatography and buffer exchanged to 50 mM MOPS (pH 7.4). GalNAc transfer from both the human and bovine B4GalT1 (Y285L/Y289L) catalyzed reactions were carried out simultaneously at 37 °C with **2** (15 mg/ml), UDP-GalNAc (10 eq), 10 mM Mn^2+^ and 5% (w/w) of enzyme: IgG. Time points were taken every hr up to 5 h and then overnight. All time points were treated with IdeS to release the Fc domain from the antibody and analyzed by LC-MS. Fc domain of GalNAc2-trastuzumab (**3**) after IdeS treatment, M = 25,553.8 Da, found: 25,555.0 Da. Following completion of the reaction the antibodies were purified by protein A affinity chromatography.

### 4.8. Sialylation of free N-glycans by the sialyltransferases

Free N-glycan was obtained from PNGase F digestion of sialylglycopeptide (SGP) [67] according to manufacture protocol. The obtained purified glycan was treated ON with MvNA, a sialidase from *Micromonospora viridifaciens* (0.1 mg/ml), and BgaA, a 1,4 galactosidase from *Streptococcus pneumoniae,* (0.1 mg/ml) to obtain desialylated degalactosylated free N-glycan (G0-GN2 in Scheme S1) and purified using porous graphitized carbon (PGC) columns after monitoring of the completion of the reaction via HPAEC-PAD and MALDI. GalNAc was transferred to degalactosylated free glycan (G0-GN2, 0.76 μmol) using UDP-GalNAc (5 eq), hB4GalT1 (0.1 mg/ml), MnCl_2_ (10 mM) in 50 mM MOPS buffer, pH 7.4, at 37 °C. Reaction was monitored using HPAEC-PAD. After completion of the reaction GalNAc2-GN2 was purified using PGC column.

Sialyltransferase reactions on free N-glycans were carried out by incubation of GalNAc2-GN2 (10 mM), CMP-Neu5Ac (50 mM), and the respective sialyltransferase (Pd2,6ST or PmST1M144D) (0.1 mg/ml) in a Tris buffer (100 mM, pH 7.5) at 37 °C. The reaction was monitored by HPAEC-PAD analysis.

### 4.9. Preparation of G2-trastuzumab-F241A (6)

Trastuzumab-F241A when expressed in HEK293T cells bore a mixture of glycoforms with varying levels of bisecting-GlcNAc, terminal Gal and Sia. To make a homogenous preparation of G2-trastuzumab-F241A (**6**), the trastuzumab-F241A (6.0 mg) was first deglycosylated to produce GNF-trastuzumab-F241A by incubating with immobilized Endo-S2 (100:1 w/w) in PBS buffer (100 mM, pH 7.4) at RT, gently shaking for 12 h. Completion of the reaction was analyzed by LC-MS by IdeS treatment to release the Fc. (M = 24,060.5 Da, found 24,060.0 Da). The deglycosylated antibody was purified by protein A affinity chromatography. Homogenous mixture of **6** was prepared by enzymatic transglycosylation by incubating GNF-Trastuzumab-F241A (5.0 mg) with G2-oxazoline (30 eq) and EndoS2 D184 M (100:1 w/w) at 30 °C for 20 to 40 min [68]. Completion of the reaction was analyzed by LC-MS by IdeS treatment to release the Fc. (M = 25,480.0 Da, found 25,480.0 Da).

### 4.10. Sialylation of intact antibodies

Transfer of Neu5Ac from CMP-Neu5Ac (150 eq) to GalNAc2-trastuzumab (**3**),G2-trastuzumab (**1**), or G2-F241A trastuzumab (**6**) (30 mg/ml) was catalyzed by Pd2,6ST/PmST1M144D (0.2 mg/ml) in Tris buffer (100 mM, pH 7.5) at 37 °C. All time points were treated with IdeS to release the Fc domain from the antibody and analyzed by LC-MS. The reaction yield was calculated as the sum of the disialylated product yield and one-half of monosialylated product yield. S1GalNAc2-Trastuzumab M = 25,926.9 Da, found: 25,928.0 Da; S2GalNAc2-Trastuzumab: M = 26,218.1 Da, found: 26,219.0 Da S1G2-Trastuzumab M = 25,844.9 Da, found: 25,845.0 Da; S2G2-Trastuzumab: M = 26,135.9 Da, found: 26,136.0 Da S1G2-Trastuzumab-F241A M = 25,771.1 Da, found: 25,771.0 Da; S2G2-Trastuzumab-F241A: M = 26,062.1 Da, found: 26,062.0 Da.

### 4.11. Expression and purification of 3F6-hIgG1

Recombinant 3F6 was produced by transient expression in HEK293T suspension cells. Cells were grown in serum-free FreeStyle^™^ F17 Expression Medium at an initial density of 8 × 10^5^ cells/mL and maintained at 37 °C with 7% CO_2_ and agitation at 150 rpm. Transient transfection was performed 20 to 24 h after seeding using 2 μg/mL plasmid DNA and 9 μg/mL polyethylenimine (PEI). The cultures were continued under the same conditions for 6 d, with supplementation of l-glucose and GlutaMAX^™^ on day 4 to support cell viability. On day 6, the cultures were clarified by centrifugation (4500 rpm, 30 min, 4 °C), and the supernatant was subjected to Protein G affinity purification. Bound antibodies were eluted with 30 mM citrate buffer (pH 2.6), immediately neutralized with 1 M Tris-HCl (pH 8.5), and buffer-exchanged into PBS (pH 7.4).

### 4.12. Enzymatic glycan remodeling of 3F6-hIgG

#### Full galactosylation of antibody 3F6.

The recombinant antibody (3F6-hIgG1) was buffer exchanged to 50 mM MOPS buffer (pH 7.4). The constituents were added at the final concentrations listed, 3F6-hIgG1 (15 mg/ml), UDP-Gal (15 eq), Mn^2+^ (10 mM). The pH of the reactions was tested to be pH 7.4. The hB4GalT1 was added last at 5% (w/w) with enzyme:IgG. Completion of the reaction was analyzed by LC-MS by IdeS treatment to release the Fc.

#### Preparation of GalNAc-terminated 3F6.

First the G0 glycoform of 3F6 (**9**) was prepared by incubating with BgaA at a final enzyme concentration of 0.1 mg/ml, ON at 37 °C. Then the G0 glycoform (**9**) (15 mg/ml) was incubated with UDP-GalNAc (10 eq), 10 mM Mn^2+^ and hB4GalT1-Y285L at 5% (w/w) of enzyme: IgG. Following completion of the reaction the antibodies were purified by protein G affinity chromatography.

### 4.13. FcγRIIIA binding analysis by ELISA

FcγRIIIA V158 (0.5 μg/mL) in PBS buffer (pH 7.4) was coated onto a high-binding 96-well plate (Santa Cruz Biotechnology) overnight at 4 °C. After washing with PBST (150 μL, 3x), the plate was blocked with 100 μL of 1% BSA (in PBST) for 2 h. After three washes with PBST, 100-μL serial dilutions of GalNAc2-Trastuzumab (**3)**, G2-F-Trastuzumab (**1**) (3000 nM- 0.0384 nM fivefold dilutions) were added to each well, followed by incubation for 1 h. After incubation, the plate was washed five times with PBST, followed by incubation with 100 μL of anti-human IgG F(ab’)2 HRP (1:5000 dilution; Invitrogen) for 1 h. Finally, after five more washings, 100 μL of substrate, 3,3′,5,5′-tetramethylbenzidine, was added for signal development. The reaction was stopped by the addition of 100 μL of 2 N sulfuric acid. Absorbance at 450 nm was measured with a Spectra MaxM5 microplate reader (Molecular Devices), and data were analyzed using three-parameter nonlinear regression.

### 4.14. C1q binding analysis by ELISA

Same steps were followed as FcγRIIIA V158 ELISA binding, except C1q (5 μg/mL) in PBS buffer (pH 7.4) was coated onto a high-binding 96-well plate (Santa Cruz Biotechnology) overnight at 4 °C. GalNAc2-3F6 (**7**), G2–3F6 (**8**) (5000 nM - 0.305 nM fourfold serial dilutions) were added to each well, followed by incubation for 1 h.

### 4.15. Siglec-2 binding analysis by ELISA

S2G2-Trastuzumab (**4**) was prepared by transferring S2G2-Oxazoline (30 eq) to GNF-Trastuzumab catalyzed by EndoS2 D184 M (1% w/w antibody: enzyme). The reaction was completed in 1 h S2GalNac2-Trastuzumab (**5**) was prepared by transferring Neu5Ac from CMP-Neu5Ac to GalNAc-F-Trastuzumab catalyzed by Pd2,6ST as described above. The reactions were pushed to obtain mostly disialylated S2GalNAc2-glycoform. Both glycoforms were purified via Protein A chromatography. Same steps were followed for FcγRIIIA V158 ELISA binding, except Siglec 2 (2 μg/mL) in PBS buffer (pH 7.4) was coated onto a high-binding 96-well plate (Santa Cruz Biotechnology) overnight at 4 °C. Antibody glycoforms **4**, **5** (22,000 nM − 10.05 nM (three-fold serial dilutions) were added to each well, followed by incubation for 1 h.

## Supplementary Material

SI

## Figures and Tables

**Table 1:** The expression levels of mammalian β-1,4-galactosyltransferases expressed in *E. coli* Origami cells.

Enzyme	Source	Expression Level
MBP_Del1_43hB4GalT1_His6	*Homo sapiens*	45 mg/L
MBP_Del1_43bB4GalT1_His6	*Bos taurus*	30 mg/L
MBP_Del1_43hB4GalT1Y285L_His6	*Homo sapiens*	40 mg/L
MBP_Del1_43bB4GalT1Y289L_His6	*Bos taurus*	20 mg/L

## Data Availability

Data will be made available on request.

## Appendix A. Supplementary data

Supplementary data to this article can be found online at https://doi.org/10.1016/j.carres.2026.110019.
