# Supplementary material for: Chemoenzymatic synthesis of unusual antibody glycoforms using E. coli expressed human and bovine β-1,4-galactosyltransferase and their mutants
Source: Carbohydr Res. Author manuscript; Available in PMC 2026 Sep 1. (PMC13439607; doi:10.1016/j.carres.2026.110019)
Supplement: Supplementary file 1 [file NIHMS2198201-supplement-SI.pdf]

Supporting Information

Chemoenzymatic synthesis of unusual antibody glycoforms using *E. coli* expressed human and bovine β-1,4-galactosyltransferase and their mutants

D. Natasha Owitipana, Darnell Harris, Helena Yun, Guanghui Zong, Lai-Xi Wang*

Department of Chemistry and Biochemistry, University of Maryland, 8051 Regents Drive,

College Park, Maryland 20742, United States

*Corresponding author. E-mail address: wang518@umd.edu

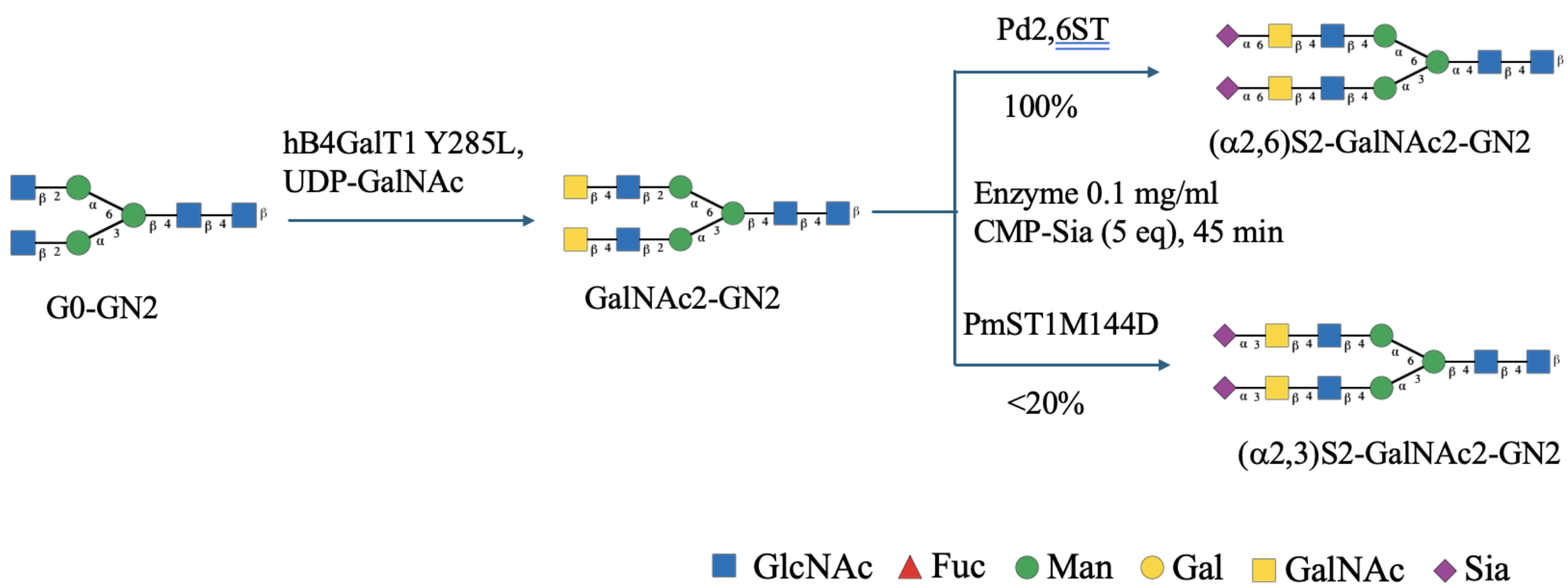

Scheme S1: Sialylation of the GalNAc terminated bi-antennary N-glycan by bacterial sialyltransferases. A solution of the GalNAc terminated N-glycan (GalNAc-GN2) (10 mM, 1eq) and CMP-Neu5Ac (50 mM, 5eq) in a buffer (100 mM, pH 7.5) was incubated with the α-2,6-sialyltransferase (Pd2,6ST) or the α-2,3-sialyltransferase (PmST1 M144D) (0.1 mg/ml) at 37 ºC. The reactions was monitored by HPAEC-PAD. The yields are shown for each enzymatic reaction at 45 min.

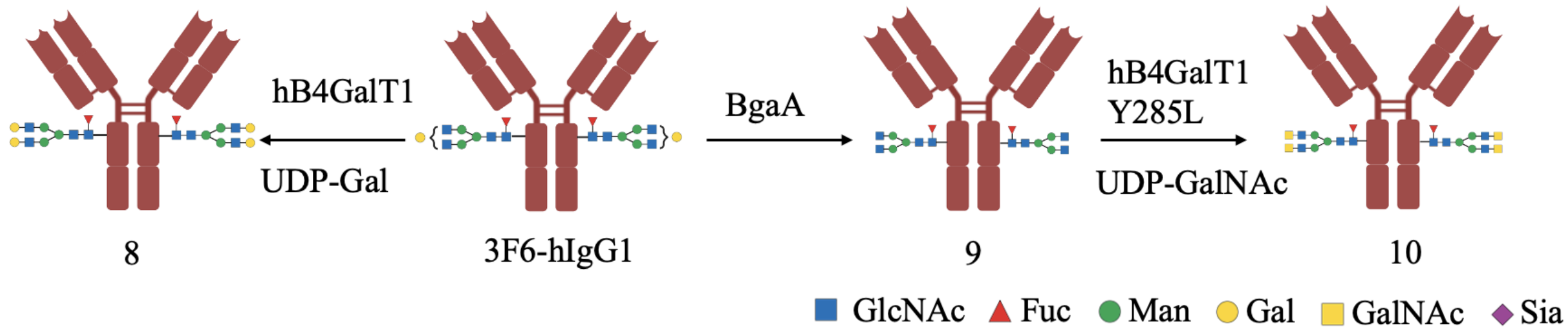

Scheme S2: Preparation of the LacdiNAc glycoform of 3F6-hIgG1 antibody by enzymatic Fc glycan remodeling. 3F6-hIgG1 was treated with β1,4 galactosidase, BgaA from *Streptococcus pneumoniae* (0.1 mg/ml) to produce the degalactosylated G0-3F6 glycoform (**9**). Then **9** (15 mg/ml) was incubated with UDP-GalNAc (10 eq) and hB4GalT1-285L (5% w/w) to produce GalNAc2-3F6 (**10**). Reactions were done at 37 °C, pH 7.4, 10 mM $MnCl_2$. Similarly, the G2 glycoform of 3F6-hIgG1 (**8**) was synthesized by incubation of 3F6-hIgG1 (15 mg/ml) with UDP-Gal (15 eq) and hB4GalT1 (5 % w/w enzyme: antibody) in a buffer (pH 7.4, containing 10 mM $MnCl_2$) at 37 °C.

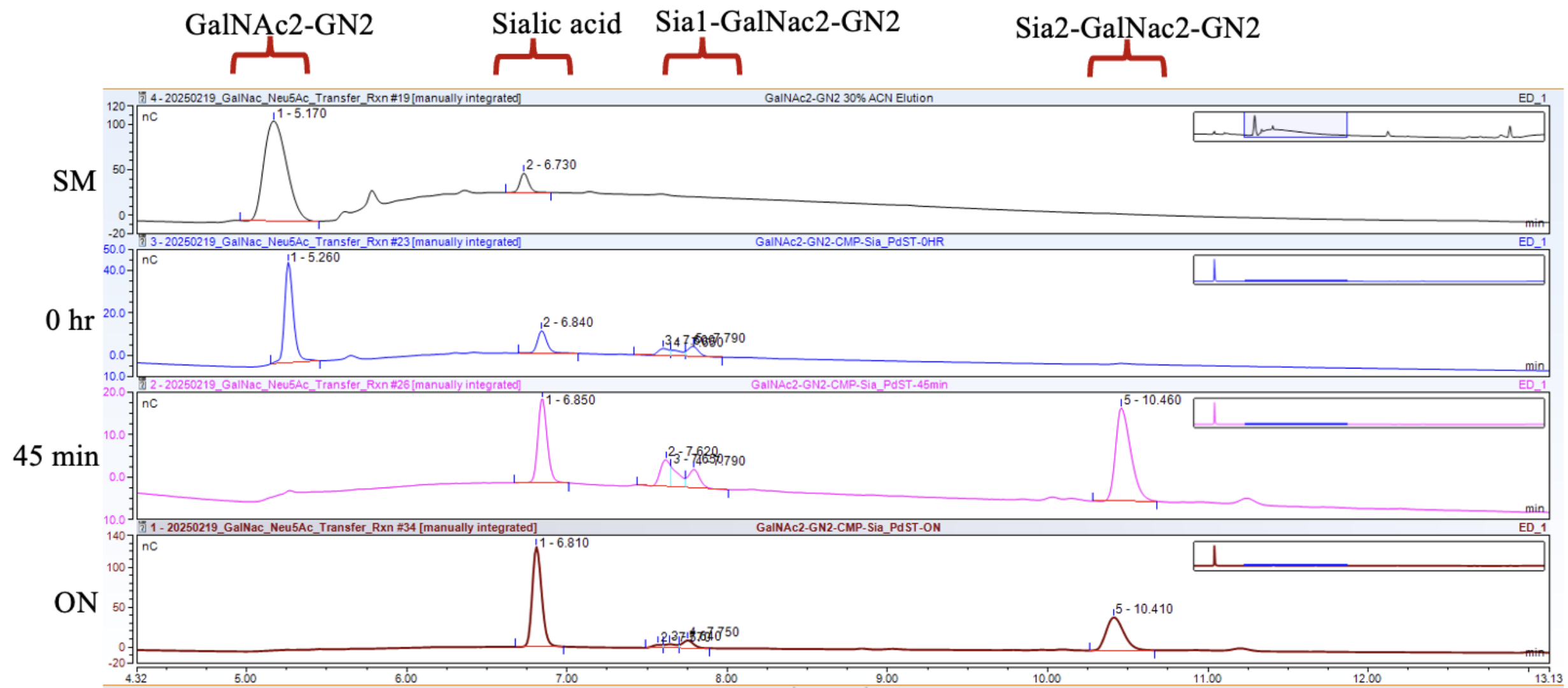

Figure S1: HPAEC-PAD monitoring of the sialylation of GalNAc2-GN2 by Pd2,6ST. Reaction conditions: CMP-Sia (50 mM, 5eq), free glycan (GalNAc2-GN2) (10 mM, 1eq), Pd2,6ST (final concentration, 0.1 mg/ml), buffer (100 mM, pH 7.5).

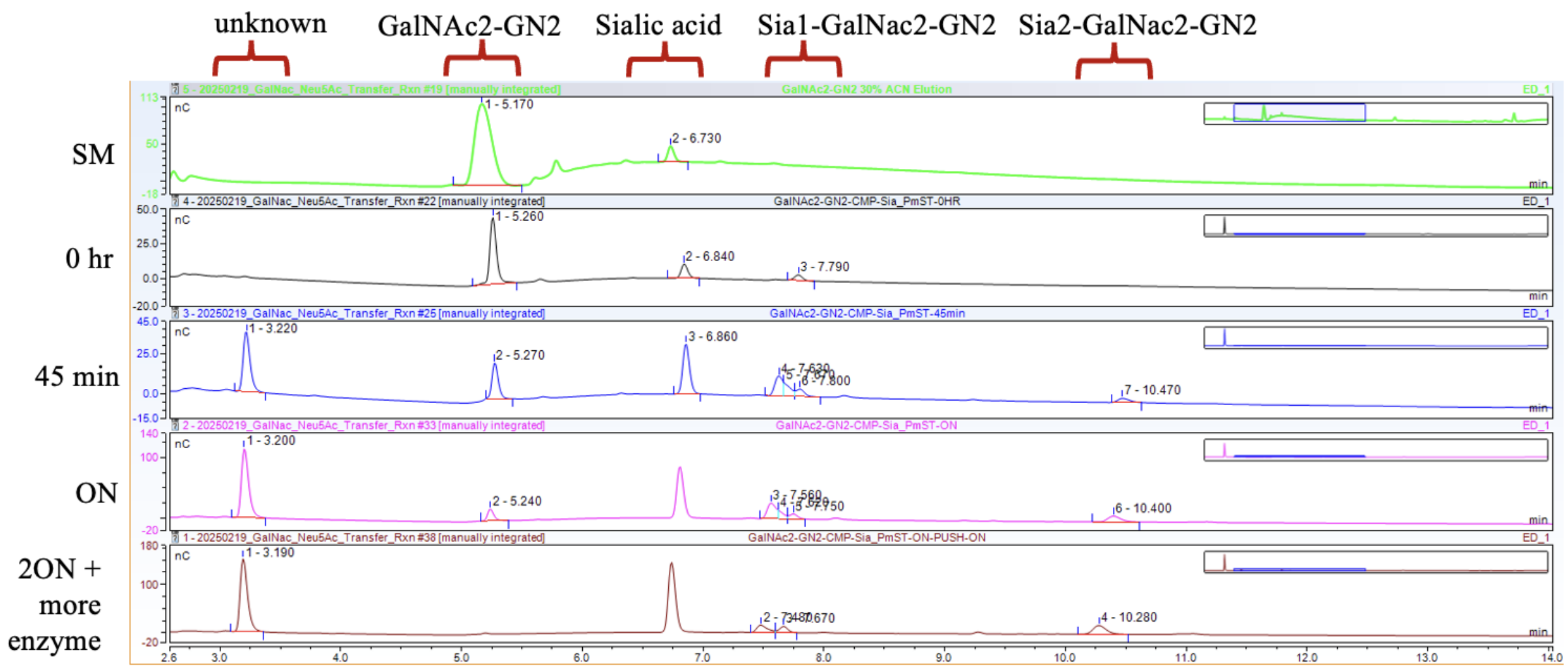

Figure S2: HPAEC-PAD monitoring of the sialylation of GalNAc2-GN2 by PmST1 M144D. Reaction conditions: CMP-Sia (50 mM, 5eq), free glycan (GalNAc2-GN2) (10 mM, 1eq), PmST1 M144D (final concentration, 0.1 mg/ml), buffer (100 mM, pH 7.5).

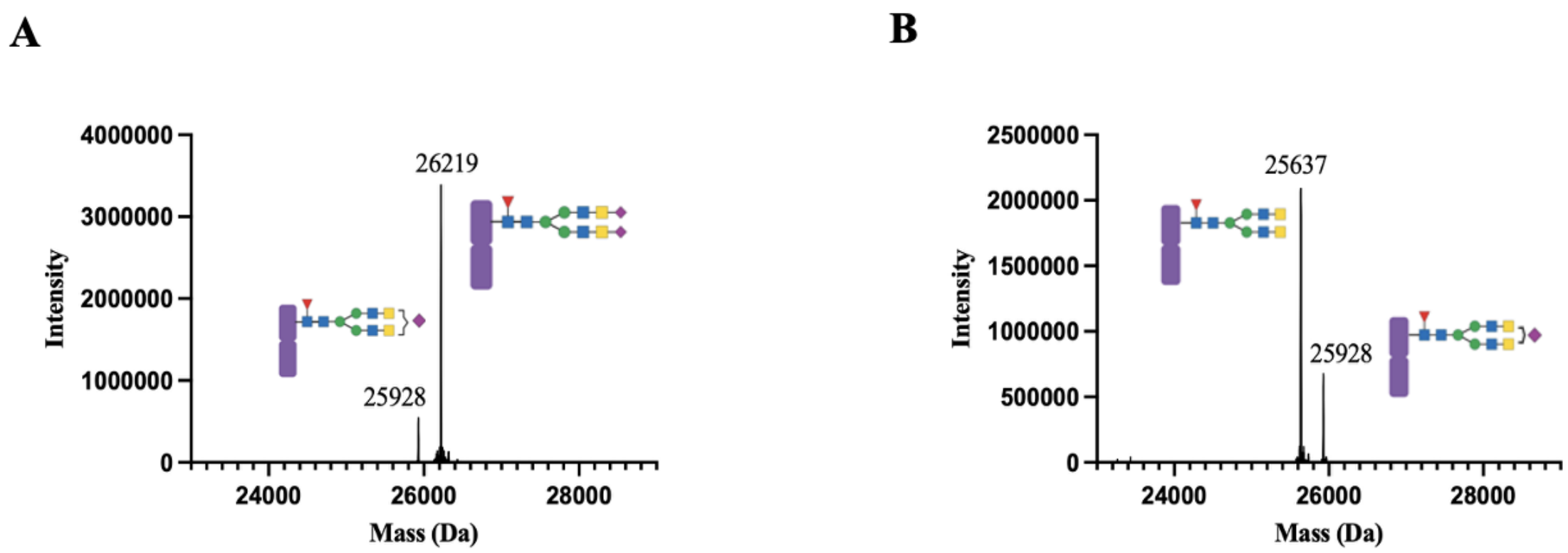

Figure S3: LC-MS analysis of sialylated LacdiNAc antibody glycoforms. Charge deconvoluted profiles are of the Fc domain after IdeS treatment. A) S2GalNAc2-trastuzumab glycoform (**5**) produced by Pd2,6ST. (S1GalNAc2-Trastuzumab expected: 25,926.9 Da, found: 25,928 Da; S2GalNAc2-Trastuzumab: expected: 26,218.1 Da, found: 26,219.0 Da) B) Glycoform **5** produced by PmST1 M144D. (GalNAc2-Trastuzumab expected: 25,635.7 Da, found: 25,637.0 Da; S1GalNAc2-Trastuzumab: expected: 25,926.9 Da, found: 25,928.0 Da)

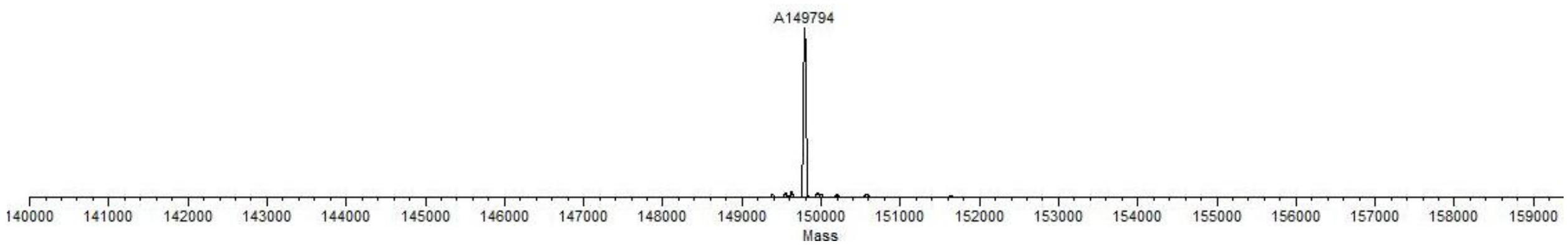

Figure S4: Charge deconvoluted LC-MS of the intact LacdiNAc glycoform of 3F6-hIgG1 (**7**).

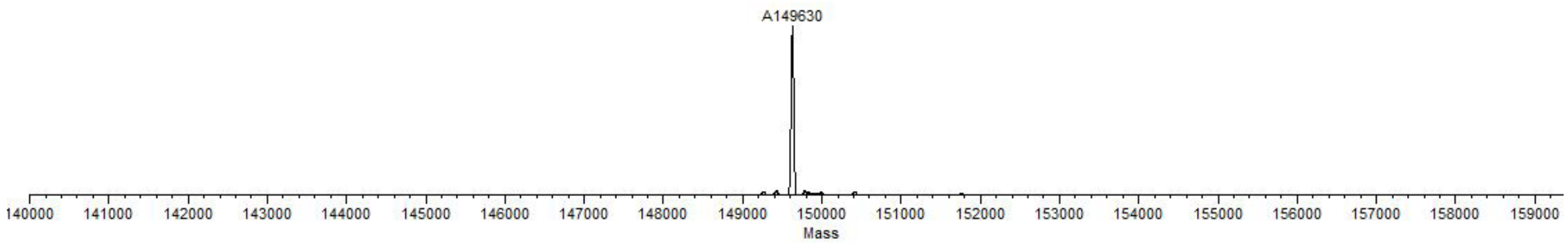

Figure S5: Charge deconvoluted LC-MS of the intact G2F glycoform of 3F6-hIgG1 (**8**).
